# Modulatory Effects of Photobiomodulation on Oxidative and Inflammatory Responses in a Murine Model of Periodontitis

**DOI:** 10.3390/antiox13121450

**Published:** 2024-11-26

**Authors:** Larissa Trarbach Figueiredo Braga, Isadora Martins Ribeiro, Maria Eduarda de Souza Barroso, Edgar Hell Kampke, Lorena Nascimento Santos Neves, Sara Cecília Andrade, Guilherme Heleodoro Barbosa, Marcella Leite Porto, Silvana Santos Meyrelles

**Affiliations:** 1 Graduate Program of Dental Sciences, Federal University of Espirito Santo (UFES), Av. Marechal Campos, 1468, Maruípe, Vitória 29043-900, ES, Brazil; 2 Graduate Program of Physiological Sciences, Federal University of Espirito Santo (UFES), Av. Marechal Campos, 1468, Maruípe, Vitória 29043-900, ES, Brazil; isadoramartins77@gmail.com (I.M.R.); m.eduarda.sb@live.com (M.E.d.S.B.); edkampke@hotmail.com (E.H.K.); losantosneves@gmail.com (L.N.S.N.); sara.andrade@edu.ufes.br (S.C.A.); guilherme.h.barbosa@edu.ufes.br (G.H.B.); 3 Laboratory of Cell Culture, Federal Institute of Espirito Santo (IFES), Av. Ministro Salgado Filho, 1000, Vila Velha 29106-010, ES, Brazil; marcella.porto@ifes.edu.br

**Keywords:** periodontitis, photobiomodulation, reactive oxygen species, oxidative stress, flow cytometry, mice, PBM

## Abstract

Periodontitis, an oral disease initiated by a dysbiotic dental biofilm, has an unclear response to photobiomodulation (PBM) as an adjunctive treatment. This study investigates the effects of PBM on reactive oxygen species (ROS), apoptosis, oxidative stress, and inflammatory markers in a periodontitis model using C57BL/6 mice, divided into four groups: control (C), control + PBM (C + PBM), periodontitis (P), and periodontitis + PBM (P + PBM). An infrared diode laser (808 nm, 133.3 J/cm^2^, 4 J/session) was applied for three days. PBM reduced superoxide anions, hydrogen peroxide, and apoptosis in gingival cells, while decreasing systemic inflammation and protein oxidation. In the P + PBM group, pro-inflammatory cytokines IL-6 and IL-12p70 decreased, whereas IL-10 increased, suggesting improvements in oxidative stress and inflammation profiles.

## 1. Introduction

Periodontitis is an oral disease characterized by a chronic immunoinflammatory reaction triggered by dysbiotic microorganisms present in dental biofilm [1]. It affects around 35% of the adult population [2], leading to irreversible loss of the tooth-supporting structures and, eventually, the teeth themselves [3]. Gingival tissue serves as the primary site for the cellular changes implicated in the pathogenesis of periodontal diseases, and dysregulation of the local inflammatory response in the gums facilitates both the onset and progression of periodontitis [4].

Evidence of alveolar bone loss [1], resulting from an uncontrolled inflammatory reaction in the periodontal tissues [3], is a critical criterion for diagnosing periodontitis. In addition to inflammation and alveolar bone loss, periodontitis is characterized by a state of oxidative stress at the cellular level [5]. This imbalance disrupts the equilibrium between anti- and pro-inflammatory mediators, leading to excessive production of free radicals, which further impairs the immune response and induces cell death [6,7].

Scaling and root planning, performed with manual or ultrasonic instruments, is considered the gold standard treatment for periodontitis. It improves clinical parameters, reduces bacterial load, limits the progression of the disease, and mitigates systemic health risks [8,9]. Scaling and root planning has the capability to diminish the bacterial load and the inflammation caused by microbial activity. Notably, while this procedure seeks to establish a suitable environment for tissue reattachment, it can also, due to its invasive aspects, traumatize the inflamed periodontal tissues, leading to discomfort during probing, as well as inflammatory mediators generated in periodontal tissues, that can enter the bloodstream, resulting in systemic effects [7].

Photobiomodulation (PBM) is a therapeutic technique that involves the application of precise doses of laser light energy, produced by red and near-infrared wavelengths within the electromagnetic spectrum, often termed the therapeutic window [8]. This photonic energy is absorbed by cytochrome C oxidase, situated on the outer membrane of the mitochondria. Similarly, it reduces the levels of inflammatory mediators such as prostaglandin E2 (PGE2), tumor necrosis factor-alpha (TNFα), cyclooxygenase-2 (COX-2), the receptor activator of nuclear factor kappa-Β ligand/osteoprotegerin ratio (RANKL/OPG), interleukin-1 beta (IL-1β), and interferon-gamma (IF-γ). This leads to improved tissue oxygen diffusion, thereby facilitating the tissue repair process [10,11,12].

Specifically, in periodontal tissues, PBM has been shown to reduce inflammation and pain [13] and to promote tissue repair following surgical procedures [14,15,16]. However, the efficacy of this therapy depends on several parameters, including the laser wavelength, the energy dose administered, and the condition of the irradiated tissue [17].

The objective of the present study was to evaluate the effects of a PBM protocol on reactive oxygen species (ROS) and apoptosis in gingival tissue, as well as on markers of systemic inflammation and oxidative stress, in an animal model of experimental periodontitis.

## 2. Materials and Methods

### 2.1. Animals

The study was conducted in accordance with ethical principles for the handling and care of animals, fully complying with international ethical standards for experimental animal research. The animals were kindly provided by the Animal Research Facility of the Federal University of Espírito Santo. The study protocol was approved by the Institutional Animal Care and Ethics Committee of the Federal University of Espíirito Santo (CEUA/UFES no. 27/2022). The sample size was determined based on Miot (2011) [18], using the standard deviation, significance level, and sampling error estimates obtained from the literature [19,20].

A total of 48 C57BL/6 mice (both sexes, aged 16 weeks, with a mean body weight of 25–30 g) were utilized in the study. The mice were housed in groups of three in polypropylene cages under controlled conditions: temperature maintained at 23 °C ± 2 °C, relative humidity at 55% ± 10%, and a standard 12 h light/dark cycle. Standard chow and water were provided ad libitum. The animals were randomly divided into four experimental groups: control (C) without periodontitis; control plus PBM (C + PBM) without periodontitis, treated with PBM; periodontitis (P) with ligature-induced periodontitis; and periodontitis plus PBM (P + PBM) with ligature-induced periodontitis treated with PBM. The control groups were essential to illustrate the absence of periodontitis and to evaluate how photobiomodulation (PBM) might influence healthy tissues. The inclusion of the control + PBM group allows us to assess the isolated effects of PBM in non-inflamed tissues, providing a clearer understanding of whether PBM induces any systemic effects in the absence of periodontal disease. This comparison helps differentiate the laser’s effects on healthy versus inflamed tissues, offering more robust conclusions.

### 2.2. Experimental Periodontitis Model and PBM Protocol

The animals were anesthetized via intraperitoneal injection of ketamine hydrochloride (91 mg/kg) and xylazine hydrochloride (9.1 mg/kg) and placed on a surgical table adapted to facilitate access to the oral cavity. Periodontitis was induced by ligation, following the method by Ribeiro et al. (2023) [21]. Briefly, a 6–0 silk thread (Ethicon, Raritan, NJ, USA) was looped around the right mandibular first molar of each animal using endodontic spacers #20 and #25 (Dentsply Maillefer, Ballaigues, Switzerland). After 28 days of periodontitis induction, the ligature was removed, and scaling and root planning (SRP) were immediately performed using manual instruments [21]. Once SRP was completed, PBM was performed. An aluminum gallium arsenide (AlGaAs) infrared diode laser (808 nm; MMOptics Ltd., São Carlos, Brazil) was applied to the extraoral region of the mandible adjacent to the right mandibular first molar for 40 s, with a total energy of 4 J/session (continuous wave mode, ø ~0.03 cm^2^, 100 mW, 133.3 J/cm^2^), on three consecutive days, for a total of three sessions. An energy density ranging from 1–5 J/cm^2^ is described as having positive effects on various cells and organs [22].

### 2.3. Sample Collection and Processing

The animals were euthanized through an overdose of ketamine and xylazine on the day following the third laser application. Blood samples were collected via cardiac puncture. Gingival tissues covering the right mandible were excised and processed with 1 mL of phosphate-buffered saline (PBS) and 2% fetal bovine serum (FBS), 0.002 g of collagenase II, and 10 µL of DNase, then homogenized and incubated for 30 min. Next, 20 µL of EDTA was added, and the mixture was incubated for 10 min, mixed with 11 mL of PBS (2% FBS), and centrifuged (400× *g*, 10 min). The supernatant was discarded, and the cells were resuspended in 2 mL of PBS (2% FBS), filtered (70 µm), recentrifuged (200× *g*, 5 min) [4], and stored for later analysis using flow cytometry to assess the levels of reactive oxygen species (ROS) and apoptosis rates. After the gingival tissue was removed, the mandibles were prepared to confirm the presence of periodontitis. The organic material was extracted from the samples by immersing them in 3% sodium hypochlorite for four weeks. Following this step, the mandibles were rinsed with distilled water. The samples were then dried in an oven at 37 °C and subsequently stored in a dry environment [21]. The collected blood was centrifuged, and 20 μL of plasma was reserved for biochemical assays to evaluate myeloperoxidase (MPO) and advanced oxidation protein product (AOPP) activity. Cytokine levels in plasma were also measured using flow cytometry.

### 2.4. Experimental Periodontitis Confirmation

To confirm the success of experimental periodontitis, a morphometric assessment of alveolar bone loss was performed on dissected and prepared mandibles. Images were digitized directly from a scanning electron microscope (SEM) (Jeol, JEM-6610 LV, Musashino, Akishima, Tokyo, Japan). The linear distance from the cementoenamel junction (CEJ) to the alveolar bone crest (ABC) of the distal molar root was measured in micrometers (μm) by an experienced blinded investigator (IMR) using public-domain Image J software 1.54j version [21]. Periodontitis was determined by the presence of 15% or more bone loss between the control and ligature groups. This criterion reflects the current classification of periodontal disease in humans and the existence of radiographic bone loss for the diagnosis of periodontitis [1].

### 2.5. Intracellular ROS Levels

The production of ROS in gingival tissue was evaluated by measuring the intracellular levels of superoxide anion (O_2_^•−^) and hydrogen peroxide (H_2_O_2_). In brief, the median intensity of fluorescence resulting from the oxidation of the markers dihydroethidium (DHE) and 2′,7′-dichlorodihydrofluorescein diacetate (DCFH-DA) was assessed using a flow cytometer (BD FACSCanto II, Becton Dickinson, São Paulo, SP, Brazil). To quantify the emitted fluorescence, cell samples were excited at 488 nm, and signals were captured using 585/42 and 530/30 filters (Becton Dickinson, São Paulo, SP, Brazil) for DHE and DCF, respectively. Data acquisition was performed using a FACSCanto II system and overlay histogram plots were analyzed using FACSDiva software version 8.0 (Becton Dickinson) to determine the average fluorescence intensity in 10,000 cells [23].

### 2.6. Gingival Cell Apoptosis

To assess apoptosis in gingival tissue, cells were labeled using the commercially available Annexin V-FITC Apoptosis Detection Kit^®^ (BD Pharmingen, San Diego, CA, USA). This kit comprises Annexin V protein conjugated to fluorescein isothiocyanate (FITC) and the vital dye propidium iodide (PI), allowing for the distinction between viable and nonviable cells. The samples were then analyzed using a flow cytometer with excitation at 488 nm, and FITC and PI fluorescence were detected using 530/30 and 585/42 filters, respectively. A total of 10,000 events per measurement were recorded. Subsequently, the data were analyzed using FACSDiva software version 8.0 and expressed as the percentage of apoptotic cells. Based on the dot plot obtained, the sum of quadrants Q2 and Q4 was determined, where Q2 represents early apoptotic cells (Annexin V positive and PI negative), and Q4 represents late apoptotic cells (Annexin V positive and PI positive). The data were expressed as the percentage of apoptotic cells [23].

### 2.7. Systemic Inflammatory Activity and Oxidative Stress Evaluation

Plasma samples were diluted in phosphate-buffered saline (PBS, 1:80) and assayed in triplicate to measure markers of systemic inflammatory activity. The MPO level was quantified as a marker of pro-inflammatory activity. In brief, plasma samples were transferred to a flat-bottom microplate, and the biochemical reaction was initiated by the addition of an o-dianisidine solution. The absorbance was measured in an iMark^®^ Absorbance microplate reader (Bio-Rad, Washington, DC, USA) at a wavelength of 460 nm, and the data were recorded at 15 s intervals over 10 min [24].

The AOPP levels, a marker of oxidative stress on proteins, were determined spectrophotometrically using the colorimetric method with chloramine T. The absorbance at 340 nm was immediately measured in an iMark^®^ Absorbance microplate reader (Bio-Rad, Washington, DC, USA). AOPP was quantified by linear regression using a standard chloramine T solution at concentrations ranging from 5 to 100 µM. The results were expressed in μM/L of chloramine T equivalent [25].

### 2.8. Serum Cytokines Quantitation

Serum cytokines interleukin 6 (IL-6), interleukin 12p70 (IL-12p70), interleukin 10 (IL-10), interferon gamma (IFN-γ), tumor necrosis factor (TNF), and monocyte chemoattractant protein 1 (MCP-1) were quantified using flow cytometry (FACSCanto II-BD) and the commercially available Cytometric Bead Array (CBA) Mouse Inflammation Kit (BD Biosciences). Bead populations were detected based on their fluorescence intensities (fluorescence detector 4 (Becton Dickinson, São Paulo, SP, Brazil), PE, wavelength ~650 nm (FL4 Red)). The fluorescence of 5000 microbeads per sample was read on forward-versus-side-scatter (FSC vs. SSC) dot plots. Prior to the analysis, a standard curve was constructed using the same procedure, allowing for the determination of cytokine concentrations in the samples of interest [26].

### 2.9. Statistical Analysis

Data were expressed as the mean ± standard error of the mean (SEM). The normality of the distribution was assessed using the Shapiro–Wilk test. Between-group comparisons were conducted using one-way analysis of variance (ANOVA). When significant differences were identified by ANOVA, Tukey’s post hoc test was employed for multiple comparisons. *p*-Values < 0.05 (*) and (#) were considered statistically significant. All analyses were performed using GraphPad Prism version 8.02 (GraphPad Inc., San Diego, CA, USA).

## 3. Results

### 3.1. Confirmation of Experimental Periodontitis Model

**Figure 1:**
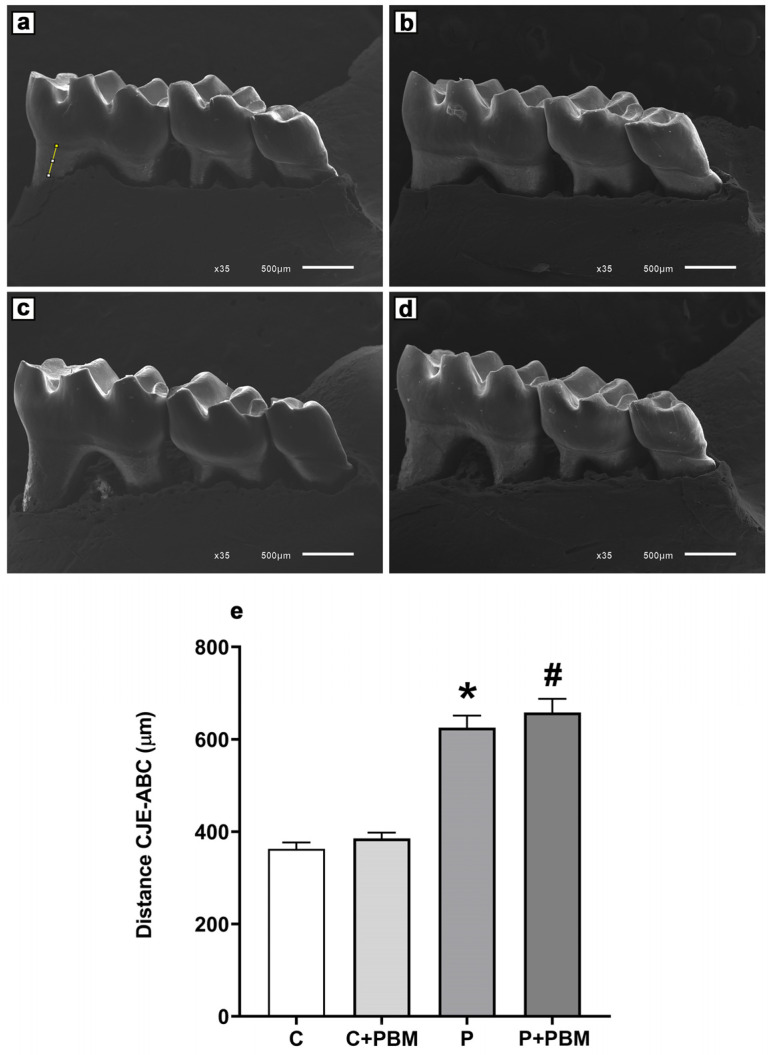
Confirmation of alveolar bone loss was made through the measurement of the distance (μm) between the cementoenamel junction (CEJ) and the alveolar bone crest (ABC) in the experimental groups (yellow line). Scanning Electron Microscopy images were acquired in direct mode (35× magnification, scale bar: 500 μm). (**a**) Group C (control); (**b**) Group C + PBM (control + PBM); (**c**) Group P (periodontitis) and (**d**) Group P + PBM (periodontitis + PBM). (**e**) Representative bar chart of alveolar bone loss (µm) in the experimental groups. The values are presented as the mean ± SEM (one-way ANOVA, Tukey’s post hoc test, n = 12). * *p* < 0.05 vs. C and C + PBM. ^#^ *p* < 0.05 vs. C and C + PBM groups.

The periodontitis experimental model was validated and analyzed through morphometric measurements of alveolar bone loss at the distal root of the mandibular molar (lingual surface). Figure 1 shows that the P group had significant alveolar bone loss (625.3 ± 26.33 µm) compared to the C and C + PBM groups (362.8 ± 13.87 µm and 385.5 ± 12.51 µm, *p* < 0.05), respectively. PBM did not prevent alveolar bone loss in the P + PBM group (658.3 ± 29.01 µm) compared to the P group, *p* > 0.05.

### 3.2. PBM Reduces ROS Production and Apoptosis in Gingival Cells

**Figure 2:**
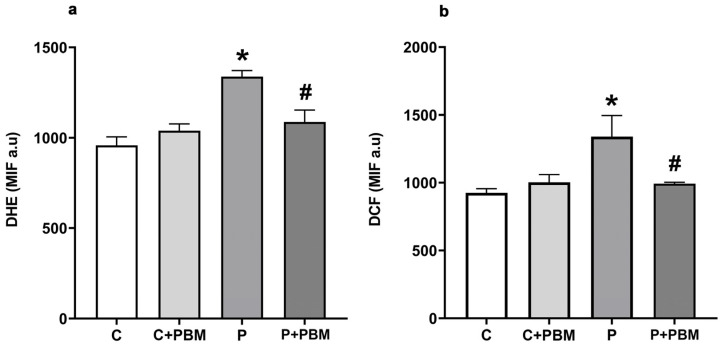
DHE (**a**) and DCF (**b**) levels in the experimental groups. The data are expressed as the median fluorescence intensity (MFI). The results are presented as the mean ± SEM (one-way ANOVA, Tukey’s post hoc test, n = 5–6). * *p* < 0.05 vs. C, C + PBM and P + PBM. ^#^ *p* < 0.05 vs. P.

To evaluate the effect of PBM on local ROS production, superoxide anion, and hydrogen peroxide levels were assessed using the fluorescence markers DHE and DCF, respectively, in gingival tissue. Figure 2a shows that the P group had an increase in superoxide production (1338 ± 33.25 MIF a.u., *p* < 0.05) compared to the C group (958.2 ± 46.76 MIF a.u.) and C + PBM group (1040 ± 372.8 MIF a.u.). PBM significantly reduced superoxide levels in the P + PBM group (1087 ± 65.52 MIF a.u.) compared to the P group, (*p* < 0.05). We observed that hydrogen peroxide levels (Figure 2b) also increased significantly in the P group (1340 ± 155.0 MIF a.u., *p* < 0.05) compared to the C group (925.0 ± 30.23 MIF a.u.) and C + PBM group (1003 ± 56.31 MIF a.u.). PBM treatment was able to prevent increases in hydrogen peroxide levels in the P + PBM group (992.8 ± 10.43 MIF a.u.) compared to the P group, *p* < 0.05.

**Figure 3:**
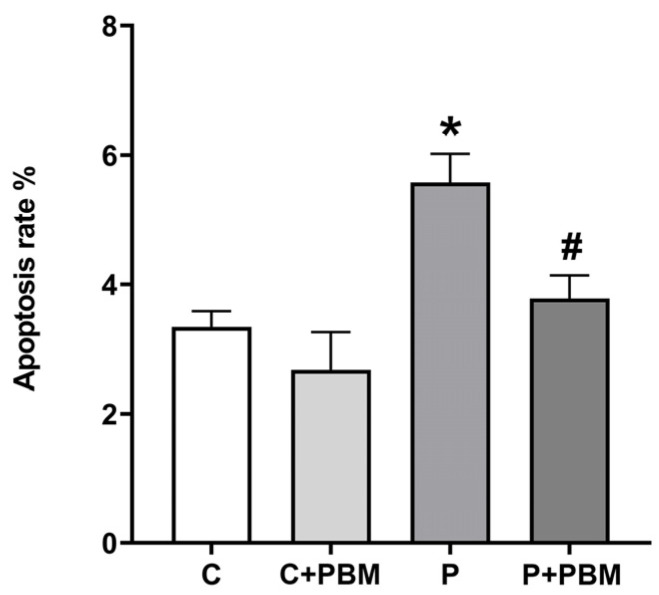
Gingival cells apoptosis rate (%) in the experimental groups. The values are presented as the mean ± SEM (one-way ANOVA, Tukey’s post hoc test, n = 5–6). * *p* < 0.05 vs. C, C + PBM, and P + PBM. ^#^ *p* < 0.05 vs. P.

The apoptosis of gingival cells was also analyzed. Figure 3 shows that periodontitis significantly increased the gingival cell apoptosis rate (5.578 ± 0.4431%, *p* < 0.05) compared to that in the C group (3.345% ± 0.2450%) and C + PBM group (2.676 ± 0.5875). PBM was very effective in reducing gingival cell apoptosis as shown in the P + PBM group (3.782 ± 0.3591, *p* < 0.05).

### 3.3. Potential Systemic Anti-Inflammatory and Antioxidant Effects of PBM

**Figure 4:**
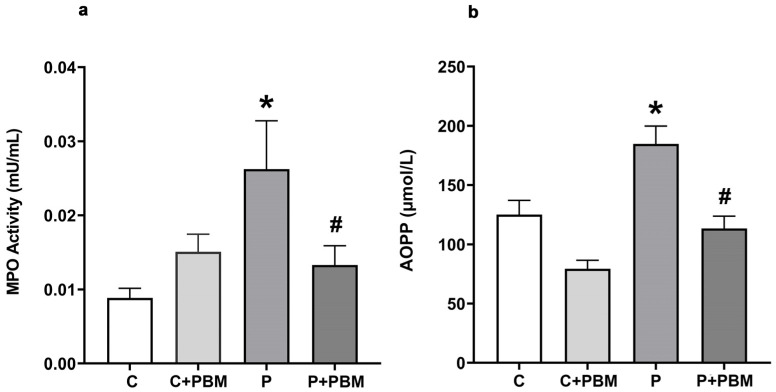
(**a**) Pro-inflammatory enzyme myeloperoxidase (MPO) plasma activity (mU/mL) and (**b**) advanced oxidation protein product (AOPP) levels in the experimental groups (µmol/L). The values are presented as the mean ± SEM (one-way ANOVA, Tukey’s post hoc test, n = 6–12). * *p* < 0.05 vs. C, C + PBM and P + PBM. ^#^ *p*< 0.05 vs. P.

To assess the impact of PBM on systemic inflammatory activity, myeloperoxidase (MPO) activity was analyzed in the plasma. MPO is a well-established biomarker of neutrophil-driven inflammation. Its elevated levels are associated with increased oxidative stress and tissue damage in various inflammatory diseases, including periodontitis [27]. The analysis of MPO allowed us to assess the systemic effects of periodontitis and PBM, extending beyond local gingival inflammation. Figure 4a shows that systemic MPO activity was significantly increased in the P group (0.02623 ± 0.006534 mU/mL, *p* < 0.05) compared to the C and C + PBM groups (0.008850 ± 0.001308 mU/mL and 0.01509 ± 0.002361 mU/mL, respectively). PBM applied to the periodontal area was very effective in decreasing MPO in the P + PBM group (0.01331 ± 0.002580 mU/mL, *p* < 0.05 vs. P group) to levels comparable to those of the control groups. Figure 4b shows the results of systemic oxidative stress production measurements in the experimental groups. Periodontitis significantly increased oxidative stress, as measured through AOPP production, in the P group (184.8 ± 15.01 mU/L, *p* < 0.05) compared to the C (125.2 ± 11.95 mU/L) and C + PBM (79.40 ± 7.167 mU/L) groups. The PBM procedure was effective in decreasing oxidative stress levels in the P + PBM group (113.5 ± 10.29 mU/L, *p* < 0.05) compared to the periodontitis group.

The plasma levels of cytokines in the experimental groups are shown in Table 1. Pro-inflammatory cytokines, IL-6 and IL-12p70, were significantly increased, while the level of the anti-inflammatory cytokine IL-10 was significantly decreased in the P group compared to C and C + PBM groups. PBM treatment significantly reversed these effects in the P + PBM group. No differences among experimental groups were observed for the IFN-γ, TNF, and MCP-1 levels.

## 4. Discussion

Our findings demonstrated that, in the experimental model used herein, photobiomodulation therapy attenuated ROS production and apoptosis in gingival tissue and reduced systemic inflammation and the pro-oxidant status. While several studies have utilized gingival tissue from experimental models of periodontitis to assess inflammatory and oxidative parameters [4,20], this is the first study to analyze the levels of superoxide anion, hydrogen peroxide, and percent apoptosis in gingival tissues affected by periodontitis, and assess the impact of an adjunctive PBM protocol. This contextualizes our work as an important research framework.

PBM is a noninvasive technique that has been investigated as an advantageous treatment option for several oral conditions, including periodontal disease [14]. Our measurement of ROS levels in gingival tissue demonstrated that PBM was able to reduce these markers compared to the untreated group. Modulating inflammatory and oxidative processes in periodontal tissues through the antioxidant potential of PBM suggests a protective effect of laser therapy against cell and tissue damage in periodontitis. This finding aligns with previous in vitro studies that detected reductions in ROS [22,28,29]. However, it is important to note that Rupel et al. (2018b) [17] reported that a wavelength lower than that used in our study does not produce any effect in cells exposed to lipopolysaccharides (LPS) and can increase ROS levels in healthy cells. Additionally, Chen et al. (2011) [30] and Wang et al. (2022) [31] reported that high laser energy densities can lead to increased ROS release. Despite the positive effects of PBM observed in experimental and in vitro studies, clinical studies demonstrating its potential to reduce oxidative stress in periodontal tissues are still lacking.

Neutrophils and macrophages produce superoxide anions as part of the defense mechanism against pathogens, and elevated levels can disrupt tissue organization in periodontitis. Our results demonstrated that PBM regulates superoxide anions, which may suggest modulation of the immune response by reducing ROS levels, thereby minimizing tissue damage and controlling chronic inflammation. As a result, periodontal tissue integrity could be preserved, contributing to the maintenance of oral homeostasis.

We observed that in the PBM-treated group, apoptosis levels remained lower than in the group of animals with periodontitis treated with basic periodontal therapy alone. The modulation of ROS decreases apoptosis, thereby facilitating the repair of affected tissues [32]. Faria et al. (2020) [33] confirmed that PBM coordinates morphological, molecular, and biochemical changes within the cell to produce a reparative effect, by regulating cell death-related proteins, which can alter the permeability of the outer mitochondrial membrane, thus modulating both the intrinsic and extrinsic pathways of apoptosis. At a time when new perspectives are emerging regarding the role of mitochondrial dysfunction in periodontitis [12,34], PBM appears to play a crucial role as an adjunct to conventional periodontal therapies. Photobiomodulation’s action on mitochondria stimulates the synthesis of adenosine triphosphate (ATP) and can influence ROS regulation in cells and tissues undergoing oxidative stress [12,30,35]. This suggests that PBM has the potential to address underlying cellular mechanisms contributing to periodontal disease and may enhance the effectiveness of conventional treatment strategies.

The laser, absorbed by mitochondria via cytochrome C oxidase, can enhance enzymatic activity by increasing oxygen consumption and stimulating adenosine triphosphate (ATP) synthesis through the photodissociation of inhibitory nitric oxide. Superoxide production in the mitochondria likely activates superoxide dismutase (SOD), converting it into hydrogen peroxide, which can cross mitochondrial membranes and activate beneficial signaling pathways. The modulation of ROS by PBM, involving anti-inflammatory mechanisms, may not be the only explanation. Other signaling pathways, such as those involving nitric oxide, cyclic adenosine monophosphate (AMP), and calcium, may also play a significant role in reducing inflammation, though further investigation is needed [36]. Additionally, PBM may influence key molecular pathways such as nuclear factor kappa-B (NF-κB), which plays a crucial role in regulating inflammatory gene expression, and nuclear factor erythroid 2-related factor 2 (Nrf2), which is responsible for upregulating antioxidant defenses. By modulating these pathways, PBM could exert both anti-inflammatory and antioxidant effects, contributing to tissue protection and repair [37,38]. This expanded view aligns with the existing literature and provides a more comprehensive understanding of PBM’s mechanisms of action in the context of periodontal inflammation.

Our investigation revealed a reduction in levels of inflammatory activity and protein oxidation, indicating the systemic anti-inflammatory and antioxidant potential of PBM in periodontitis. Experimental studies examining the effect of PBM on MPO activity in periodontitis have reported findings like ours [21,27,39]. Increased AOPP levels typically signify advanced oxidative stress and are linked to various systemic chronic inflammatory conditions [40]. Additionally, previous research by Rupel and Ottaviani (2018a) [41] demonstrated a reduction in AOPP levels in the gingival crevicular fluid (GCF) of patients undergoing periodontal treatment with adjunctive PBM. These findings underscore the potential systemic benefits of PBM as an adjunctive therapy for periodontitis.

We observed a modulating effect of PBM on pro-inflammatory and anti-inflammatory cytokines, characterized by a reduction in IL-6 and IL-12p70 and an increase in IL-10 levels. Additionally, we noted a significant difference in the IL-6/IL-10 ratio, indicating a potential systemic anti-inflammatory effect in our experimental model. However, cytokines such as IFN-γ and TNF-α, which are also involved in periodontitis development and are recognized in the literature for their pro-inflammatory profile [42], did not show significant changes. This finding could be attributed to species-specific responses, as animal models may exhibit differences from humans due to the complex bacterial load associated with the disease.

It is important to acknowledge that the inflammatory response in periodontitis is multifaceted, involving numerous mediators. The lack of significant changes in IFN-γ and TNF-α does not necessarily reflect PBM’s ineffectiveness; rather, it may indicate that PBM’s mechanisms of action do not target these specific cytokines. One possible explanation is that T helper 1 (Th1) cells produce IFN-γ and TNF-α, which activate macrophages and stimulate IgG2a production, mediating a macrophage-dominant host defense response [43].

This reduction in pro-inflammatory cytokine levels provides further evidence of the role of PBM in modulating inflammation. PBM can promote modulation in key cells of the inflammatory response, such as neutrophils, macrophages, and lymphocytes, by inhibiting pro-inflammatory signaling pathways, regulating oxidative stress and ROS production, and enhancing blood microcirculation and tissue oxygenation. Through these modifications, PBM can alter the inflammatory profile and response. Previous in vitro studies [44,45] have demonstrated the beneficial regulatory effect of PBM on cytokine secretion, shifting it from a pro-inflammatory to an anti-inflammatory profile. However, clinical studies have reported divergent results regarding the effect of PBM on cytokine levels in the context of periodontal treatment with different application protocols. While some studies have reported findings like ours [46], others have described an absence of effect of PBM on cytokine regulation [47,48], underscoring the need for further research.

The results we obtained in the periodontitis group align with findings from previous studies [12,27,34] regarding the mechanisms of oxidative stress involved in the progression of periodontal disease. Increased ROS levels, including hydrogen peroxide, and oxidation, contribute to mitochondrial dysfunction [12], cell death [49], and, clinically, periodontal attachment loss [27]. Oxidative stress also entails systemic pro-inflammatory cytokines such as IL-6 and IL-12 [50].

The PBM protocol utilized in our study achieved a steady state for some of the parameters of interest. However, the challenge of achieving an optimal anti-inflammatory dose and stimulating physiological homeostasis remains a weakness of PBM therapy [51], given its biphasic dose–response relationship [35] and its ability to alter host defense mechanisms dependent on dose, exposure time, and energy intensity [17,35]. A limitation of this study is that the methodology employed, with euthanasia conducted one day after the final laser application, did not allow for the evaluation of the potential long-term therapeutic effects of the laser. However, based on the beneficial effects observed in gingival cells, future investigations are needed to elucidate the effects of this PBM protocol on bone repair in the context of experimental periodontitis as well. To address this limitation, future studies should extend the observation period to evaluate the sustained impact of PBM on both tissue regeneration, bone, and the inflammatory response. Additionally, different experimental protocols, with varied application frequencies and dosages could help optimize PBM’s long-term benefits. Previous clinical studies have also indicated the need for prolonged evaluations, as bone repair outcomes have not been consistently observed despite extended laser use [46,47,48]. Incorporating longer follow-up periods in both preclinical and clinical studies is crucial to better understand the therapeutic potential of PBM in managing periodontitis over time. Our findings provide further evidence of the promising role of PBM as an adjunct to conventional periodontal therapy. PBM has the potential to mitigate both local and systemic inflammatory and oxidative processes involved in the pathogenesis of periodontitis.

## 5. Conclusions

In the experimental model of periodontitis used in this study, photobiomodulation (PBM) demonstrated a beneficial effect on key mechanisms involved in the development and progression of periodontal disease. We observed reductions in reactive oxygen species and apoptosis in gingival cells, as well as improvements in systemic parameters, suggesting a potential systemic and local benefit of PBM. The anti-inflammatory and antioxidant properties identified in this study highlight the promise of PBM as an adjunctive therapy in periodontal treatment. However, while our preclinical findings are promising, further clinical validation is essential. Future clinical trials are needed to replicate these results in human patients with periodontitis, confirming both the local and systemic effects of PBM in a clinical setting. Additionally, the research should aim to further elucidate the underlying mechanisms of PBM’s effects to optimize its use in periodontal care and potentially expand its applications in broader therapeutic contexts.

## Figures and Tables

**Table 1:** Plasma cytokine levels in the experimental groups.

Groups/Parameters	C	C + PBM	P	P + PBM
IL-6	5.49 ± 0.29	5.83 ± 0.25	6.99 ± 0.27 *	4.66 ± 0.22 ^#^
IL-12p70	16.94 ± 1.63	15.62 ± 0.31	24.36 ± 1.32 *	18.54 ± 0.93 ^#^
IL-10	20.57 ± 0.84	20.46 ± 0.90	17.83 ± 0.30 *	23.17 ± 0.36 ^#^
IFN-γ	2.00 ± 0.08	2.06 ± 0.25	2.23 ± 0.16	2.33 ± 0.30
TNF	17.70 ± 0.42	15.20 ± 0.33	16.68 ± 0.77	16.97 ± 0.70
MCP-1	48.83 ± 1.28	46.69 ± 2.35	55.61 ± 5.61	50.83 ± 0.73
IL-6/IL-10	0.25 ± 0.02	0.27 ± 0.00	0.39 ± 0.02 *	0.20 ± 0.01 ^#^

Data are expressed as the mean ± SEM (pg/mL). One-way ANOVA followed by Tukey’s post hoc test, n = 3–5. * *p* < 0.05 vs. C, C + PBM, and P + PBM. ^#^ *p* < 0.05 vs. P.

## Data Availability

Data are contained within the article.
